# The Association of Serum Irisin with Bone Mineral Density and Turnover Markers in New-Onset Type 2 Diabetic Patients

**DOI:** 10.1155/2022/7808393

**Published:** 2022-02-28

**Authors:** Xiujing Wang, Tianxiao Hu, Yun Ruan, Jiaqi Yao, Huiling Shen, Yao Xu, Bojing Zheng, Zhengying Zhang, Jing Wang, Qingying Tan

**Affiliations:** ^1^Department of Endocrinology, The 903rd Hospital of PLA, Hangzhou, China; ^2^College of Life Science, Zhejiang Chinese Medical University, Hangzhou, China

## Abstract

**Background:**

Irisin, an exercise-induced myokine and adipocytokine, has been reported to decrease in type 2 diabetic patients. Recently, several research studies indicated that circulating levels were correlated with bone mineral density (BMD). To evaluate bone metabolism, bone turnover markers (BTMs) should be included. However, with respect to newly diagnosed T2DM patients, the relevance of their irisin levels to their BTMs and BMD remains unclear. The investigation of serum irisin levels in patients who have been newly diagnosed with type 2 diabetes and illumination of the relationship between serum irisin levels and those two indices of BMD and BTMs mentioned above are the intention of this cross-sectional study.

**Methods:**

66 new-onset type 2 diabetic patients (T2DM group), together with 82 control subjects (NGT group), were recruited in this study. Serum irisin concentrations and BTMs (including osteocalcin (OC), procollagen type 1 N-terminal propeptide (P1NP), and *β*-C-terminal telopeptides of type I collagen (*β*-CTX)) were determined by the enzyme-linked immunosorbent assay (ELISA). Glucose, lipid profile, and insulin were considered as measuring indicators as well. Dual-energy X-ray absorptiometry (DXA) was utilized to evaluate the indicator of BMD. Serum irisin, BTMs, and BMD were compared between diabetic patients and healthy individuals. Pearson and Spearman correlation analyses were applied as well to assess correlations between irisin and BTMs and BMD. Multiple stepwise regression analysis was conducted to identify the independent factors of irisin. ROC curve analyses were carried out for serum irisin prediction for osteoporosis/osteopenia (OP).

**Results:**

The serum levels of irisin, procollagen type 1, intact N-terminal propeptide (P1NP), and osteocalcin (OC) were evidently lower in T2DM subjects than in NGT subjects (10.90 ± 1.88 vs .11.69 ± 2.06 ng/mL, *P* < 0.05; 36.42(25.68,51.70) vs. 44.52(35.73,58.05)ng/ml, *P* < 0.05; 16.15(12.40,21.66) vs. 18.70(15.56, 23.22)ng/ml, *P* < 0.05). Among patients with T2DM, the circulating irisin level of those with OP was lower than that of normal BMD (9.98 ± 2.09 vs. 11.39 ± 1.57 ng/ml, *P* < 0.01); irisin had a negative correlation with *β*-C-terminal telopeptides of type I collagen (*β*-CTX) (*r* = −0.496, *P* < 0.001) and came back unrelated to Lumbar BMD; Lumbar BMD was negatively relevant to OC (*r* = −0.274, *P* < 0.05) and *β*-CTX (*r* = −0.410, *P* < 0.01). Multiple linear regression analyses of stepwise models implied that TG, LDL-C, and *β*-CTX were independently associated with serum irisin concentrations (*P* < 0.01 or *P* < 0.05).

**Conclusion:**

Serum irisin level was declined in patients with type 2 diabetes diagnosed in the near term and had a certain association with bone turnover markers. It is suggested to consider irisin as a potential biomarker of bone metabolic disorder in T2DM patients with the initial diagnosis.

## 1. Introduction

Skeletal muscle (SKM) and bone are anatomically and physiologically connected, and their coupling has been considered mainly a mechanical one to regulate bone metabolism. Besides, SKM can affect bone homeostasis also in a nonmechanical manner, through a network of molecules termed myokines [1, 2], which were produced and released by myocytes, cleaved from fibronectin type III domain-containing protein (FNDC5). Residing in one of the representatives in the myokine family, irisin is mainly known for the transiting ability of white adipocytes to brown adipocytes [3]. Most studies about this myokine were focused on the field of energy expenditure and adiposity or glucose homeostasis whereas recent pieces of evidence implied that irisin was also involved in bone metabolism. Studies showed that, to some extent, circulating irisin is related to osteoporotic fractures in women experiencing postmenopausal periods [4, 5]. The plasma irisin standard has a positive bearing on bone mineral status in populations of different ages [6–8]. Besides, Colaianni et al. [9] reported that, compared with myoblasts of housed nonexercised mice, those mice subjected to 3 weeks of uncontrolled wheel running augmented their irisin expression; the conditioned media (CM) collected from myoblasts of exercised mice induced osteoblast differentiation in vitro in a larger measure than the well-rested house mice. Lately, injection of recombinant irisin (r-irisin) was reported to increase osteoblasts and decrease the quantity of osteoclasts, which evokes the upkeep of quality and bone mass in male mice [10] and ovariectomized mice [11]. All these highlighted that irisin had an underlying function as a biomarker or modulator of bone abnormalities in the T2DM patient group where they are more susceptible to fracture which was suggested to be considered one of the chronic complications of T2DM [12–14].

Low bone mineral density (namely, BMD) could cause a possible fracture effect on patients; apart from that, studies have shown that T2DM subjects were also characterized by normal or increased BMD compared to sex, age, and BMI matched controls [14, 15]. Therefore, BMD may underestimate the possibility of fracture occurring inside patients with T2DM [16]. It was reported that low bone turnover may be one of the factors contributing to increased bone fragility in diabetes [17, 18]. The role of irisin in bone metabolism in patients with T2DM remains unclear. Several studies have revealed that the expression of irisin was decreased in T2DM patients in comparison with control subjects [19, 20]; however, some others reported elevated irisin levels in T2DM patients [21, 22]. Whether there is a relationship of irisin with BMD and BTMs in newly diagnosed T2DM patients remains unknown. In this cross-sectional study, we aim to identify the serum irisin levels in the new-onset T2DM patients and control subjects and do some investigation to confirm the existence of associations between irisin levels and the BMD and BTMs in both groups.

## 2. Materials and Methods

### 2.1. Research Objects

Comprising 66 newly diagnosed T2DM patients (T2DM group, mean age 51.08 ± 12.67 years) and 82 healthy controls whose age and sex are matched (NGT group, mean age 50.04 ± 8.98 years) according to 75 g OGTT outcomes, a total amount of 148 subjects were recruited from the outpatient and inpatient of the endocrinology department of the 903rd Hospital of PLA, China, from May 2017 to December 2019. Those subjects were distributed into four subgroups in accordance with BMD levels [normal BMD subjects (−1.0 < T/Z < 1.0) and decreased BMD subjects (T/Z ≤ −1.0, including osteopenia and osteoporosis)] for further analysis. Exclusion criteria from the study included the following: (1) subjects aged ≤18 and ≥ 70 years; (2) enduring any kind of acute infection; (3) with a fracture history; (4) heart, liver, or kidney failure; (5) hypo- or hyperparathyroidism and hypo- or hyperthyroidism; (6) pregnancy; and (7) taking medications that could interfere with bone and muscle, including corticosteroid, immunosuppressives, interferon, nonsteroidal during the recent 6 months, or other major diseases. This study has acquired the approval of the Ethics Committee of Chinese 903rd Hospital of PLA, abiding by the principles of the Helsinki Declaration on Human Experimentation. Informed consent in written form from the subjects has been obtained as well.

### 2.2. Anthropometric and Biochemical Parameters

Weight, height, hip circumference, and waist circumference, along with blood pressure [systolic blood pressure (SBP) and diastolic blood pressure (DBP)], of both groups were measured in line with a standardized protocol. Body mass index (BMI) was figured by dividing weight (kg) by height squared (m^2^); waist-hip ratio (WHR) was calculated by dividing waist circumference (cm) by hip circumference (cm). After overnight fasting for at least 10 h, blood samples were obtained. Then, plasma samples were kept at −80°C until further assayed. Total triglyceride (TG), total cholesterol (TC), low-density lipoprotein cholesterol (LDL-C), high-density lipoprotein cholesterol (HDL-C), fasting blood glucose (FBG), and 2-hour plasma glucose (2hPG) were determined by an automatic biochemical analyzer (Abbott, AerosetC16000, USA). Fasting insulin (FINS) was tested with the application of an automatic chemiluminescence immunoassay analyzer (Abbott, I4000, USA). Glycosylated hemoglobin (HbA1c) was tested by AfinionAS100 glycosylated hemoglobin analyzer (Alere, Afinion AS100, USA). The homeostasis model assessment (HOMA) method was utilized to assess insulin resistance (HOMA-IR) and *β*-cell function (HOMA-*β*) according to the equations as follows: HOMA-IR = FBG(mmol/L) × FIns(*μ*U/mL)/22.5; HOMA-*β* = 20 × FINS(*μ*U/mL)/[FBG(mmol/L) − 3.5].

### 2.3. Measurements of Irisin, Bone Turnover Markers (BTMs), and BMD

Irisin levels in serum were identified by enzyme-linked immunosorbent assay (ELISA) (Westang, Shanghai, China). Serum osteocalcin (OC), procollagen type1 intact N-terminal propeptide (P1NP), *β*-C-terminal telopeptide of type I collagen (*β*-CTX), and 25(OH)D were determined via chemiluminescence immunoassay (CLIA) according to manufacturers' instructions (Beckman, USA). BMD of the L1–L4 vertebrae (Lumbar BMD) was taken with meterage via dual-energy X-ray absorptiometry (DXA) (GE Medical systems lunar, USA) and presented with absolute values (g/cm^2^) and T-score or Z-scores.

### 2.4. Statistical Analysis

Delivered as means ± SD, the data for continuous variables of the normal distribution are subsequently analyzed with independent samples *t*-test. The data with a result of not conforming to normal distribution were expressed as *M*[P25, P75]. Through the Mann–Whitney test, the data were analyzed. The comparison of subgroups was performed using one-way ANOVA with post hoc analysis behind. As for calculating the internal relevance of irisin level and parametric and nonparametric variables, what came into application were Pearson and Spearman correlation analyses. Between irisin and variables, their independent associations were determined when multiple linear regression was taken into usage. What was calculated is the receiver operator characteristic (ROC) curve, which was utilized to confirm whether irisin possesses the ability to predict the risk of osteopenia or osteoporosis (OP). Statistically, a *P* value of 0.05 was of vital magnitude. With SPSS version 25.0 software, all statistical analyses were performed.

## 3. Results

### 3.1. Comparison of Clinical Parameters and Serum Irisin Levels of the Two Groups

**Figure 1:**
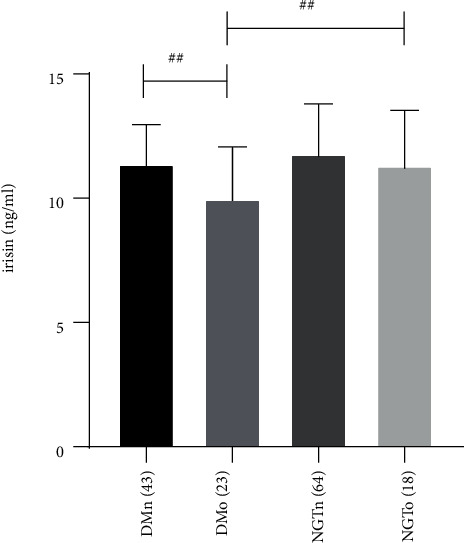
Serum irisin levels in four subgroups of DMn (T2DM with normal BMD), DMo (T2DM with osteopenia or osteoporosis), NGTn (NGT with normal BMD), and NGTo (NGT with osteopenia or osteoporosis). The number of subjects was shown in parentheses. *P* values are evaluated by a one-way ANOVA test. ^##^*P* < 0.01.

Clinical parameters, irisin levels, and BMD of the 148 subjects are shown in Table 1. Sex, age, and BMI were comparable between the two groups. In line with anticipation, T2DM subjects had higher DBP, WHR, FBG, 2hPG, HbA1c, TC, TG, and HOMA-IR and lower HDL-C and HOMA-*β* than subjects of the NGTs (*P* < 0.01 or *P* < 0.05). Importantly, the serum levels of procollagen type 1 intact N-terminal propeptide (P1NP), osteocalcin (OC), and irisin were notably lower in T2DM subjects than in NGT ones (36.42 (25.68,51.70) vs. 44.52 (35.73,58.05) ng/ml, *P* < 0.05; 16.15 (12.40,21.66) vs. 18.70 (15.56, 23.22) ng/ml, *P* < 0.05; 10.90 ± 1.88 vs .11.69 ± 2.06 ng/mL,*P* < 0.05). However, no exceedingly explicit disparities were detected in FINS, LDL-C, *β*-CTX, and Lumbar BMD in the comparable range of the two groups of subjects (*P* > 0.05). The ratio of osteopenia/osteoporosis (OP) patients was prominently higher within T2DM patients compared with the control subjects (34.8% vs. 21.9%, *P* *<* 0.01). Moreover, subgroup analysis indicated that in the T2DM group, serum irisin levels were remarkably lower in patients with OP (DMo) than in patients with normal BMD (DMn) subjects (9.98 ± 2.09 vs. 11.39 ± 1.57 ng/ml, *P* < 0.01), but no such distinction was found between NGT with OP (NGTo) and NGT with normal BMD (NGTn) subjects (11.31 ± 2.23 vs. 11.79 ± 2.01 ng/ml, *P* > 0.05), which is displayed in Figure 1.

### 3.2. Correlation of Irisin Levels and Lumbar BMD with Clinical Parameters

In both groups, the variable of Lumbar BMD is positively relevant to height and weight while negatively relevant to age. In the T2DM group, Lumbar BMD was correlated with *β*-CTX (*r* = −0.410, *P* < 0.01) and OC (*r* = −0.274, *P* < 0.05) negatively. Serum irisin concentrations were positively correlated with waist circumference (*r* = 0.336, *P* < 0.01), weight (*r* = 0.330, *P* < 0.01), BMI (*r* = 0.338, *P* < 0.01), TC (*r* = 0.244, *P* < 0.01), LDL-C (*r* = 0.388, *P* < 0.01), TG (*r* = 0.189, *P* < 0.05), and HbA1c (*r* = 0.147, *P* < 0.05) (Table 2), negatively relevant to *β*-CTX(*r* = −0.496, *P* < 0.01), but we could not observe such correlations in NGT group. Multiple linear regression analyses of stepwise models implied TG, LDL-C, and *β*-CTX were associated with serum irisin concentrations independently (*P* < 0.01 or *P* < 0.05), with the regression equation of *Y*(irisin) = 10.134 + 0.189 × TG + 0.566 × LDL-C-2.701 × *β*-CTX (*R*^2^ = 0.387).

### 3.3. The Effect of Serum Irisin on the Prediction of Osteopenia/Osteoporosis (OP)

**Figure 2:**
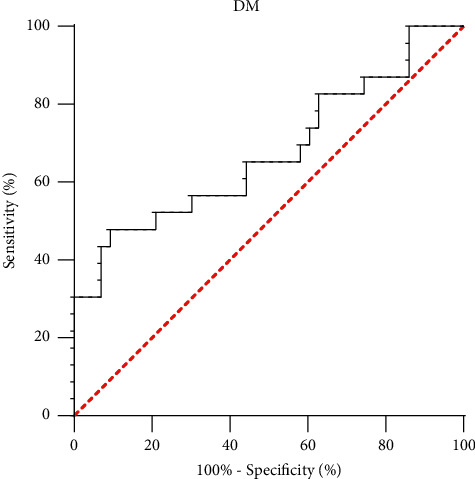
ROC curve analysis of the prediction power of serum irisin for osteopenia or osteoporosis (OP).

ROC curve analysis indicated that it is the areas under the curve (AUCs) of serum irisin that expressed the number of 0.675 for the prediction of OP in the T2DM group, while in the NGT group, no such prediction power of irisin was revealed (Figure 2).

## 4. Discussion

The cross-sectional study at present mainly revealed that the circulating levels of irisin, P1NP, and OC were lower in new-onset T2DM patients than in NGT subjects. The ratio of patients with OP was drastically higher in T2DM patients in comparison with the control subjects (34.8% vs. 21.9%, *P* *<* 0.01). Subgroup analysis indicated that in the T2DM group, patients with OP had decreased circulating irisin concentrations in contrast with those possessing normal BMD. Additionally, for people living with the disease of T2DM, Lumbar BMD was negatively correlated with *β*-CTX and OC. Waist circumference, weight, BMI, and HbA1c were positively correlated, while *β*-CTX was negatively correlated with serum irisin levels. However, irisin did not show significant associations with BMD in both groups.

The result of this study was in agreement with most studies, which mainly demonstrated that, compared with the NGT group, the levels of serum irisin had curtailed inside the T2DM patients [19, 20, 23]. However, there were other studies expounding the fact that irisin levels could elevate in T2DM subjects [21, 22]. Besides, researchers also found that irisin was positively correlated with BMI (*P* = 0.04), body fat percentage (*P* = 0.03), HbA1c (*P* = 0.03) [22], and fasting insulin (*r* = 0.303, *P* = 0.021) [24], yet negatively correlated with diabetes duration (*r* = 0.384, *P* = 0.002) [24]; partly similar observations were found in our study. Moreover, diabetes mellitus was reported with an incremental risk of hip fractures [12]. Jiajue et al. [17] found that both the diabetes group and impaired fasting glucose (IFG) group patients had decreased levels of P1NP and *β*-CTX when comparing postmenopausal Chinese women to the normal glucose group. Similarly, Song et al. [18] reported that newly diagnosed patients with T2DM have lower levels of P1NP, CTX, OC, PTH, 25(OH)D, and higher levels of bone alkaline phosphatase (B-ALP) compared to the nondiabetes group. Besides, in a hospital-based cross-sectional study which enrolled 1499 T2DM patients, Zhao et al. [25] found that OC, P1NP, and *β*-CTX had a negative relevance with Lumbar BMD in both men and women. All the above suggested that T2DM patients presented suppressed bone turnover, which might be a reasonable explanation for the increased fracture risk, independent of BMD.

Several studies were conducted to identify the relationship between BMD and irisin in different populations. Colaianni et al. [8] previously reported a positive relevance between BMD and serum irisin in children in a healthy state. The same relevance was also found in young adults regularly playing sports and older adult subjects [26–28]. By analyzing patients according to T-scores, it was reported that patients with osteopenia/osteoporosis (OP) had lower levels of irisin compared to healthy controls in matched conditions of sex and age [28]. Palermo et al. [29] revealed an interplay between irisin and PTH; they reported myotubes treated with PTH (1-34) had decreased FNDC5 expression and r-irisin administration led to a 50% downregulation of parathyroid hormone receptor (PTH-r) mRNA expression compared to untreated cells (*P* < 0.001); they also found that irisin levels were prominently inclined to a lower direction in the hyperparathyroidism (PHPT) group, rather than the age and BMI matched controls (*P* < 0.001) but no apparent bidirectional and internal BMD-irisin correlation was observed. In old type 2 diabetic patients, Martins and Aranha [30] found that PTH has a positive relevance to OC and CTX (*r* = 0.639, *P* < 0.001; *r* = 0.542, *P* < 0.001, respectively) and inversely related to BMD (*P* < 0.05). Similarly, we found that patients with OP had decreased circulating irisin levels compared to those with normal BMD in the T2DM group. Besides, we also observed that irisin was negatively associated with *β*-CTX in the T2DM group. However, between Lumbar BMD and irisin, no evidently intensive correlation has been found in both groups.

Physical exercise is a critical influential factor countering the maintenance of healthy bone metabolism. Irisin, the myokine induced by exercise has a cross talk between muscle and skeleton development [31]. Colaianni et al. [9] reported that exercising mice had higher levels of irisin levels than the housed resting mice. They also observed that the differentiation of osteoblasts was enhanced by the conditioned medium (CM) from exercised myoblasts. Moreover, improvement of cortical mineral density, bone bending strength, and geometrical architecture along with a reduction in osteoclasts was reported to be associated with injection of recombinant irisin (r-irisin) at a smaller amount of dosage [10]. Also, Zhang et al. [32] used r-irisin to treat a couple of osteoblast cell types and suggested that r-irisin had anabolic actions on the regulation of osteoblast lineage cells through aerobic glycolysis. On the contrary, Kim et al. [33] discovered that what irisin could do is to stimulate the expression of sclerostin in vitro in a dose-dependent demeanor, indicating that the activity of osteoblast was suppressed. Besides, Vadala et al. [34] found irisin increased human osteoarthritic chondrocytes (hOAC) cell content and stimulated the expression of type II collagen gene while it downregulated the expression of inducible nitric oxide synthase (iNOS) gene and decreased interleukin (IL)-1, IL-6, matrix metalloproteinase (MMP)-1, and MMP-13 in vitro, suggesting that irisin may stimulate hOAC anabolism and inhibit catabolism of it, which implied a bidirectional and internal muscle-cartilage cross talk. Clinical studies have indicated irisin to have an inverse relation with osteoporosis and fracture independently of BMD, body composition, and exercise in postmenopausal females [4, 35]. In patients on maintenance hemodialysis, serum irisin level was reported to have a positive association with BMD [36]. But Palermo et al. [35] reported that, at any site, there was no significant correlation between BMD and irisin, and serum levels of irisin were not correlated with the daily physical activity in overweight patients impacted by at least one vertebral osteoporotic fracture. All these findings indicated that irisin had fundamental effects on bone metabolism maybe as an anabolic regulator or a catabolic inhibitor, highlighting that irisin may potentially act as a mediator or a marker of bone health. However, how irisin implements a role in bone metabolism in T2DM still requires further research.

Several limitations of this study would be elaborated on in the following content. First, there was an objective flaw of the cross-sectional design, which was that the design itself failed to supply information on prospective alterations about cause-and-effect relationships. Second, the data about daily activity and other bone turnovers (including PTH and B-ALP) of subjects were not available. In addition, only BMD of lumbar 1–4 was measured; BMD of other sites (including femoral neck and total femur) was not available, leading to the failure to assess the relationship of irisin with BMD of other sites. Finally, the small sample size lessened the statistical power of this study.

In conclusion, irisin did not show a significant association with BMD in newly diagnosed T2DM patients. This study implied that the serum levels of irisin of patients living with T2DM were decreased compared to NGT subjects. In new-onset T2DM patients, circulating irisin was lower in OP subjects than that of normal BMD and was inversely correlated with *β*-CTX. These findings suggested that the myokine of irisin may serve as a messenger in muscle-bone cross talk and might be considered as a potential biomarker for bone metabolic disorder in newly diagnosed type 2 diabetic patients. The underlying mechanisms require further confirmation in specifically designed in future studies.

## Figures and Tables

**Table 1:** Clinical parameters of controls and T2DM subjects.

	NGT (*n* = 82)	T2DM (*n* = 66)	*t*/*χ*^2^/*Z*	*P*
Gender, M/F	54/28	46/20	0.246	0.620
Age (years)	50.04 ± 8.98	51.08 ± 12.67	−0.562	0.575
SBP (mmHg)	126 (114,136)	123 (120,136)	−0.261	0.794
DBP (mmHg)	76 (69,87)	80 (75,88)	−2.118	0.034^*∗*^
Weight (kg)	69.01 ± 11.75	70.84 ± 14.18	−0.859	0.392
Height (cm)	166.54 ± 6.45	165.61 ± 8.46	0.760	0.449
BMI (kg/m^2^)	24.79 ± 3.25	25.69 ± 4.22	−1.479	0.141
Waist circumference (cm)	85.80 ± 9.22	89.07 ± 11.02	−1.965	0.051
Hip circumference (cm)	94.13 ± 5.46	92.45 ± 11.91	1.064	0.290
WHR	0.91 (0.87,0.96)	0.95 (0.91,1.00)	−3.586	<0.001^*∗*^
HbA1c (%)	5.4 (5.3,5.6)	8.0 (6.3,11.0)	−9.716	<0.001^*∗*^
FBG (mmol/L)	4.94 (4.64,5.31)	8.22 (5.92,12.69)	−8.608	<0.001^*∗*^
2hPG (mmol/L)	5.78 ± 1.22	16.38 ± 5.98	−14.165	<0.001^*∗*^
FINS (uIU/ml)	6.6 (4.8,9.4)	7.7 (4.5,11.0)	−1.468	0.142
HOMA-IR	1.45 (1.09,2.22)	2.90 (1,73,5.53)	−5.736	<0.001^*∗*^
HOMA-*β*	96.94 (62.98,147.71)	36.18 (14.46,78.26)	−6.743	<0.001^*∗*^
TC (mmol/L)	4.90 ± 1.01	5.44 ± 1.40	−2.681	0.008^*∗*^
TG (mmol/L)	1.32 (0.98,2.14)	1.71 (1.14,3.11)	−3.075	0.002^*∗*^
LDL-C (mmol/L)	2.77 ± 0.83	2.85 ± 1.06	−0.537	0.592
HDL-C (mmol/L)	1.26 (1.11,1.49)	1.09 (1.00,1.24)	−3.761	<0.001^*∗*^
25(OH)D (ng/ml)	21.19 (15.66,27.01)	18.32 (14.33,23.18)	−1.832	0.067
Lumbar BMD (g/cm^2^)	1.12 ± 0.14	1.09 ± 0.16	1.151	0.251
P1NP (ng/ml)	44.52 (35.73,58.05)	36.42 (25.68,51.70)	−2.567	0.010^*∗*^
OC (ng/ml)	18.70 (15.56, 23.22)	16.15 (12.40,21.66)	−2.345	0.019^*∗*^
*β*-CTX (ng/L)	0.47 (0.33,0.65)	0.43 (0.26,0.59)	−0.172	0.864
Irisin (ng/ml)	11.69 ± 2.06	10.90 ± 1.88	2.402	0.018^*∗*^

Values were given as means ± SD or median with interquartile range. NGT, normal glucose tolerance; T2DM, type 2 diabetes mellitus; SBP, systolic blood pressure; DBP, diastolic blood pressure; BMI, body mass index; WHR, waist-hip ratio; HbA1c, glycosylated hemoglobin A1c; FBG, fasting plasma glucose; 2hPG,2-hour plasma glucose; FIN, fasting insulin; HOMA-IR, homeostasis model assessment of insulin resistance; HOMA-*β*, homeostasis model assessment of *β*-cell function; TC, total cholesterol; TG, triglyceride, LDL-C, low-density lipoprotein; HDL-C, high-density lipoprotein; Lumbar BMD, body mineral density of the L1–L4 vertebrae. P1NP, procollagen type 1 intact N-terminal propeptide; OC, osteocalcin; *β*-CTX, *β*-C-terminal telopeptides of type I collagen.

**Table 2:** Correlations of serum irisin and BMD with clinical characteristics in the two groups.

		NGT		T2DM	
		*r*	*P*	*r*	*P*
*Irisin*					
vs	Age (years)	0.180	0.106	−0.150	0.231
vs	Waist circumference (cm)	0.127	0.255	0.336	0.006^*∗*^
vs	Weight (kg)	0.096	0.391	0.330	0.007^*∗*^
vs	BMI (kg/m^2^)	0.072	0.522	0.338	0.006^*∗*^
vs	SBP (mmHg)	0.241	0.029^*∗*^	0.054	0.524
vs	HbA1c (%)	−0.005	0.964	0.167	0.049^*∗*^
vs	TC (mmol/L)	0.125	0.262	0.244	0.004^*∗*^
vs	TG (mmol/L)	0.046	0.683	0.189	0.025^*∗*^
vs	LDL-C (mmol/L)	0.145	0.195	0.388	0.001^*∗*^
vs	P1NP (ng/ml)	−0.006	0.956	−0.201	0.105
vs	OC (ng/ml)	0.123	0.272	−0.143	0.251
vs	*β*-CTX (ng/L)	0.004	0.968	−0.496	<0.001^*∗*^
vs	25(OH)D (ng/ml)	0.081	0.467	0.044	0.724
vs	Lumbar BMD (g/cm^2^)	0.038	0.736	0.189	0.129

*Lumbar BMD*					
vs	Age (years)	−0.248	0.025^*∗*^	−0.209	0.015^*∗*^
vs	Height (cm)	0.223	0.040^*∗*^	0.314	<0.001^*∗*^
vs	BMI (kg/m^2^)	0.208	0.060	0.230	0.064
vs	Weight (kg)	0.263	0.017^*∗*^	0.394	0.001^*∗*^
vs	LDL-C (mmol/L)	0.096	0.389	0.279	0.015^*∗*^
vs	P1NP (ng/ml)	−0.247	0.025^*∗*^	−0.160	0.200
vs	OC (ng/ml)	−0.224	0.053	−0.274	0.026^*∗*^
vs	*β*-CTX (ng/L)	−0.202	0.069	−0.410	0.001^*∗*^
vs	25(OH)D (ng/ml)	−0.019	0.869	0.094	0.454

## Data Availability

The datasets of the current study are available from the corresponding author upon reasonable request.
